# Idiopathic neonatal hemoperitoneum presented as scrotal hematoma: it’s a diagnostic challenge?

**DOI:** 10.1186/s13052-021-01161-x

**Published:** 2021-10-12

**Authors:** Alessia Salatto, Flavia Indrio, Vittoria Campanella, Cosetta Maggipinto, Raffaella Cocomazzi, Francesco Canale, Maria Nobili, Gianfranco Maffei, Fabio Bartoli

**Affiliations:** 1 Department of Medical and Surgical Science University of Foggia, Pediatric Surgery Ospedali Riuniti Foggia, Foggia, Italy; 2 Department of Pediatric Surgery, AUO “Ospedali Riuniti”, Foggia, Italy; 3 Department of Neonatology, AUO “Ospedali Riuniti”, Foggia, Italy

**Keywords:** Hemoperitoneum, Anaemia, Scrotal hematoma, Laparotomy

## Abstract

**Background:**

Idiopathic hemoperitoneum in the newborn is an entity very rarely encountered in clinical practice.

**Case presentation:**

A case of scrotal hemorrhage (SH) associated with intrabdominal hemorrhaging and acute anemia is presented. Indications for early surgery included a massive scrotal hematoma, rapid onset of severe anemia, and unknown etiology.

**Conclusion:**

Clinical and diagnostic approaches in a case of neonatal scrotal hematoma should be given careful consideration as abdominal in origin, and a pre-operative computed tomography (CT) scan or magnetic resonance image (MRI) in addition to an abdominal/scrotal ultrasound should be added as part of the diagnostic work-up.

## Background

Idiopathic hemoperitoneum (IH) is a rare condition in newborns that is generally due to rupture of the spleen [1, 2]. Also, a scrotal hematoma (SH) in the neonate, although a rare condition, warrants prompt diagnosis and surgical intervention [3–8]. SH commonly results from testicular torsion, adrenal hemorrhage (NAH), and birth-related trauma. However, in some cases, the cause may not be discernible, and surgical exploration be negative [6]. .Generally, this condition does not warrant early surgery before a complete diagnosis is made. We present a case of neonatal acute SH, which was found to be secondary to IH.

## Case report

A baby boy with vertex presentation was born with vaginal delivery associated to the Kristeller maneuver at 39 weeks of gestation. Obstetric history was significant for maternal trauma due to an accidental fall down the stairs at 37 weeks of gestation. An accurate prenatal ultrasound did not show trauma-related lesions or abnormalities.

**Fig. 1:**
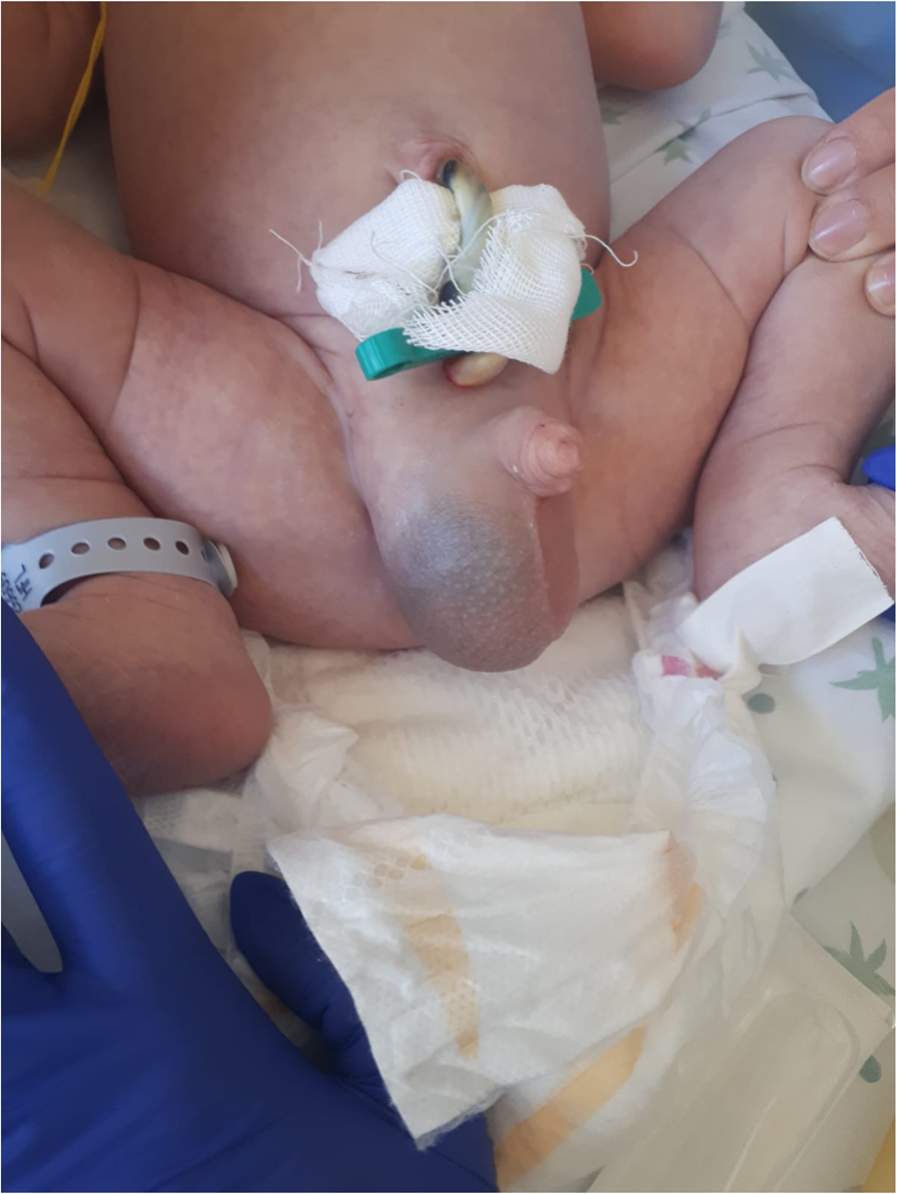
Right scrotal swelling with bluish discoloration 5 h after birth

**Fig. 2:**
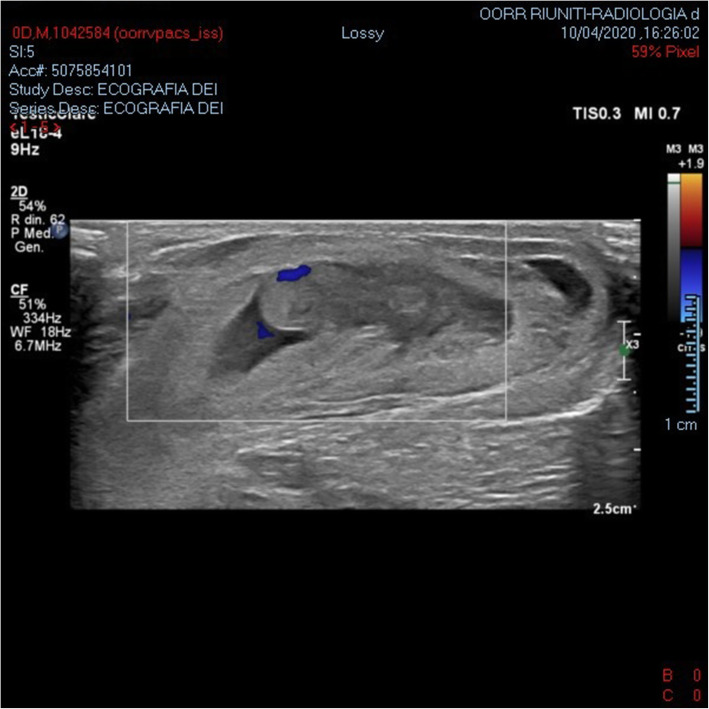
Clear vascular spots are seen on the Echo color Doppler image

Newborn birth weight was 3470 g. Apgar score was 8/9 and 9/9 at 1 and 5 min, respectively. At birth, the newborn presented an initial right hydrocele. Right scrotal swelling with bluish discoloration (Fig. 1) was noted 5 h after birth. All of the differential diagnostic procedure were performed in order to exclude strangulated hernia, NAH, and testicular torsion. Abdominal and testicular ultrasound with a Doppler study showed a right enlarged testicle (14 mm diameter) enveloped in dense hyperechoic tissue, presence of a blood hypoechoic layer, mild ascites, and no NAH. The color Doppler showed clear vascular spots (Fig. 2) that confirmed blood flow. The initial diagnosis was idiopathic SH.

Seven hours after birth, the general condition of the child suddenly worsened with the onset of paleness, blood pressure decrease, and development of tachycardia. For these reasons, he was admitted to the neonatal intensive care unit (NICU).

Physical examination revealed poor general appearance, hypoactive pupils, icteric sclera, a globular and not very treatable abdomen, and bluish discoloration of the right hemi scrotum. Vital signs indicate a pulse of 155/min, respiration 38/min, body temperature 36.7 °C, and blood pressure 60/40 mmHg. The X-ray was performed during orthostasis before the child underwent surgery and showed moderate abdominal distension without clear hydro-air levels and the absence of a subdiaphragmatic sickle and non-empty rectal ampulla. Laboratory tests showed pH 7.1, hemoglobin 8.2 g/dL, hematocrit 25.4%, white blood cell count of 28,280/mm^3^, and platelet count of 282,000/mm^3^.

Total and direct bilirubin levels were 10.71 and 0.43 mg/dL, respectively. Other signs were normal. Prothrombin time and activated partial thromboplastin time were normal. A blood transfusion of 50 mL of concentrated red blood cells was sufficient to stabilize the patient. Although the neonate was stabilized and diagnostic workup completed, the cause of bleeding was still unknown; a scrotal origin was suspected, indicating the need for surgical exploration. The laparoscopic approach in neonates is routinely used at our institution but not for scrotal disease (scrotal disease was our pre-operative diagnosis). A written consent for surgery was obtained from parents. Under general anesthesia, an inguinal incision was made to explore spermatic vessels and testis. We found severe adhesion between spermatic and surrounding tissues; however, this adhesion could not be explained by the recent bleeding episode. Also, the peritoneal vaginal duct was filed with red clotted blood while the testis appeared structure and perfused normally. After the cause of scrotal bleeding could not be found, we decided to extend the inguinal incision and reached the right iliac fossa through a subcutaneous dissection. At this point, the fascia was opened, and an hemoperitoneum was drained. At the same time, all intestinal segments, epigastric vessels, liver margins, and surrounding structures were explored; however, no cause of bleeding could be found. After drainage, the bleeding appeared completely stopped, and it was then decided not to proceed with a xifo-pubic laparotomy to further investigate the origin of bleeding. In fact, the modern approach to abdominal blunt trauma recommends not proceeding with the extended laparotomy if bleeding has stopped and hemodynamic parameters are stable.

**Fig. 3:**
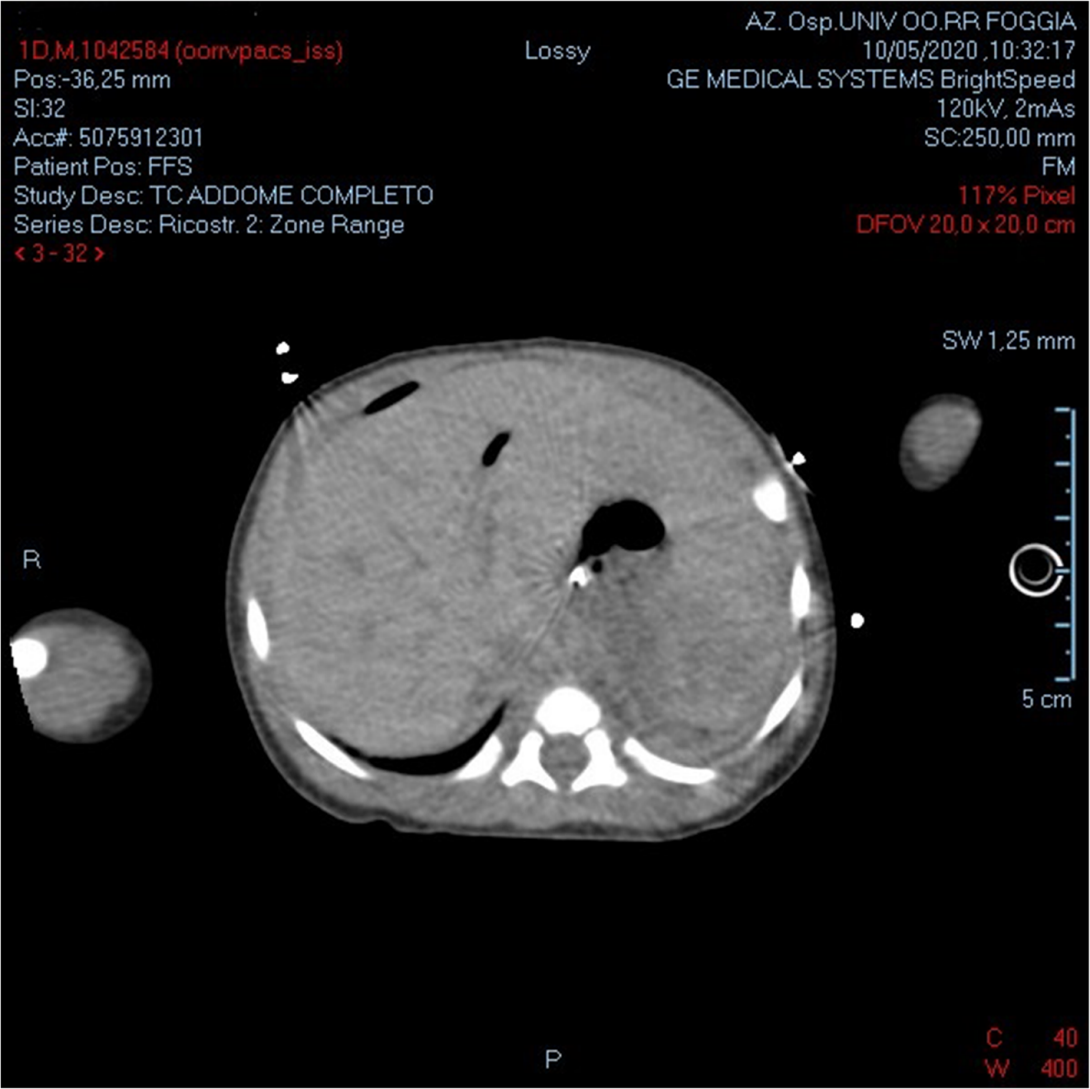
An abdominal computed tomography (CT) with contrast shows some bloody collection in the subphrenic space and between the splenic and renal veins

The following day, in consideration of the unknown origin of bleeding and lack of useful information offered by abdominal ultrasound (clearly operator-dependent), we performed an abdominal computed tomography (CT) scan with contrast that showed some bloody collection in the subphrenic space and between splenic and renal vein (Fig. 3). An abdominal CT was preferred by parents because this procedure did not require additional anesthesia. The scrotal area, on the other hand, was normal. The postoperative course was uneventful. The neonate was discharged to home on the seventh post-operative day following a normal testicular and abdominal ultrasound and blood test value with the diagnosis of IH. On the 2-week follow-up ultrasound, minimal persistence of a hematoma between renal and splenic vessels was detected. The complete coagulative profile of the neonate and his parents were normal.

The newborn was evaluated 2 months after discharge at which time the abdominal and testicular ultrasounds were normal with no sign of residual liquid. Growth parameters of the baby were normal.

## Discussion

Intra-abdominal bleeding in the newborn is uncommon [1, 2]. Also, idiopathic SH is a rare condition [3–5]. The two associated conditions and severe anemia have rarely been reported. Our case was an unusual combination of the two conditions associated with an acute severe anemia. Scrotal swelling with/without bluish discoloration in newborns can arise from hydrocele, inguinal hernia, orchitis, meconium peritonitis, testicular trauma, a testicular tumor, and/or testicular torsion [7]. Intra-abdominal hemorrhaging resulting from perinatal asphyxia, sepsis, and coagulation abnormalities can also be causes SH [8]. In our case, based on diagnostic and laboratory test results, we excluded all of the previous conditions. Testicular torsion was excluded immediately after the physical examination and ultrasound, which had been performed immediately after birth.

In addition, a differential diagnosis, including hemorrhagic disease of the newborn, sepsis with disseminated intravascular coagulation, and a solid organ injury (adrenal injury, liver and a splenic injury) was performed. However, we still were unable to make a pre-operative diagnosis. This finding confirmed that SH may prove to be a very difficult diagnostic challenge. Also, the post-operative CT scan did not clearly define the cause of the SH.

Adrenal hemorrhage in the newborn is one of the most frequent causes of intraperitoneal hemorrhaging and although it is an uncommon entity, SH is an extremely rare manifestation of NAH [9, 10]. Adrenal bleeding resulting in SH involves both testes and can be easily diagnosed using an abdominal ultrasound. Inclusion of NAH of the newborn in the differential diagnosis of an acute scrotum can prevent unnecessary surgical explorations [11, 12]. In our case, however, the surgical intervention was performed under emergency conditions due to the onset of anemia and unknown etiology.

In our case, the accidental trauma during the last 2 weeks before delivery most likely provoked a partial vascular lesion of unknown origin. This partial injury was made worse by the vaginal dystocia delivery that caused bleeding into the abdominal cavity and scrotum. Several papers have shown an increase in the incidence of birth injury with the use of forceps or vacuum assistance during delivery [13]. Pignotti et al. reported on a full-term infant born by vacuum extractor and Kristeller maneuver who had a similar, but more severe, presentation with unresponsive hemorrhagic shock who eventually died before surgery. It is well known that direct or indirect trauma during delivery may cause solid organ or vascular injury [14, 15]. In the literature, the difficulties of establishing a pre-operative diagnosis in cases with only SH as a presentation that often require urgent surgical exploration of the scrotum have been reported [6]. In fact, in those cases, a scrotal or inguinal surgical approach is justified. On the contrary, the most common causes of scrotal hematomas, such as NAH, are often managed conservatively [16]. Furthermore, the management of a self-limited hemoperitoneum is a common finding in abdominal trauma and does not justify surgical exploration in the absence of hemodynamic instability [6]. For this reason, the observation that the hemoperitoneum was self-limited during surgery justified the decision not to continue further with a more extensive surgical approach. This surgical strategy may seem risky upon initial evaluation; however, it turned to be safe and effective for the management of this neonate.

## Conclusion

Acute SH in the neonatal period is a rare condition that requires prompt diagnosis and possible urgent intervention with a more limited surgical approach. Clinical and diagnostic approaches toward a neonatal scrotal hematoma should receive careful consideration as abdominal in origin and pre-operative CT scan or MRI in addition to abdominal/scrotal ultrasound should be added as part of the diagnostic work-up.

SH most commonly occur because of testicular torsion, adrenal hemorrhage, birth trauma, and/or thrombocytopenia. However, if an SH occurs in the first few hours after delivery, a high suspicion of trauma-related delivery should be considered.

SH could underlie a more severe condition, such as hemoperitoneum with life threatening events, which requires immediate surgical intervention. A self-limiting hemoperitoneum should be managed conservatively.

Careful evaluation of any reported trauma during pregnancy could prevent acute condition, such as an intrabdominal hemorrhage leading to SH associated with severe anemia.

## Data Availability

Not applicable.

## Abbreviations

IH: Idiopathic hemoperitoneum
SH: Scrotal hematoma
NAH: Adrenal hemorrhage
