# Correction to: Idiopathic neonatal hemoperitoneum presented as scrotal hematoma: it’s a diagnostic challenge?

**DOI:** 10.1186/s13052-022-01212-x

**Published:** 2022-01-31

**Authors:** Alessia Salatto, Flavia Indrio, Vittoria Campanella, Cosetta Maggipinto, Raffaella Cocomazzi, Francesco Canale, Maria Nobili, Gianfranco Maffei, Fabio Bartoli

**Affiliations:** 1 Department of Medical and Surgical Science University of Foggia, Pediatric Surgery Ospedali Riuniti Foggia, Foggia, Italy; 2 Department of Medical and Surgical Science University of Foggia, Pediatric Surgery Ospedali Riuniti Foggia, Foggia, Italy; 3 Department of Pediatric Surgery, AUO “Ospedali Riuniti”, Foggia, Italy; 4 Department of Neonatology, AUO “Ospedali Riuniti”, Foggia, Italy

**Correction: Ital J Pediatr 47, 207 (2021)**

**https://doi.org/10.1186/s13052-021-01161-x**

The original article [1] mistakenly inverted each author’s name; this has since been corrected.
